# Refractive Index Sensing Properties of Metal–Dielectric Yurt Tetramer Metasurface

**DOI:** 10.3390/nano15201570

**Published:** 2025-10-15

**Authors:** Shuqi Lv, Paerhatijiang Tuersun, Shuyuan Li, Meng Wang, Bojun Pu

**Affiliations:** Xinjiang Key Laboratory of Luminescence Minerals and Optical Functional Materials, School of Physics and Electronic Engineering, Xinjiang Normal University, Urumqi 830054, China; shuqi0156@163.com (S.L.); wangmeng091999@163.com (M.W.); 17716902615@163.com (B.P.)

**Keywords:** metal–dielectric tetramer metasurface, refractive index sensing, quasi-bound states in the continuum, surface plasmon resonance, multiband resonance

## Abstract

The metal–dielectric hybrid tetramer metasurface has received a lot of attention in the field of optical sensing owing to the excellent refractive index sensing performance. However, achieving simultaneous high-quality *Q*-factor, polarization insensitivity, multi-band tunability across visible to near-infrared spectra, and ultra-narrow linewidth is an urgent problem to be solved. To overcome this challenge, we proposed a metal–dielectric yurt tetramer metasurface. The finite-difference time-domain method was used to simulate the sensing properties. We explored the physical mechanism of different resonance modes, optimized the structure parameters of the metasurface, and investigated the influence of incident light and environmental parameters on the sensing properties. The results show that the proposed structure not only possesses a high *Q*-factor but also exhibits excellent wavelength tunability in the visible to near-infrared band and has polarization insensitivity. By skillfully introducing the structural size perturbation, the surface plasmon resonance mode and two Fano resonance modes are successfully excited at the wavelengths of 737.43 nm, 808.99 nm, and 939.50 nm. The light–matter interaction at the Fano resonance frequencies is highly enhanced so that a maximum refractive index sensitivity, figures of merit (*FOM*), and *Q*-factor of 500.94 nm/RIU, 491.12 RIU^−1^, and 793.13 are obtained. The narrowest full width at half maximum (FWHM) is 1.02 nm, respectively. This work provides a theoretical basis for the realization of a high-performance metasurface refractive index sensor.

## 1. Introduction

**Figure 1:**
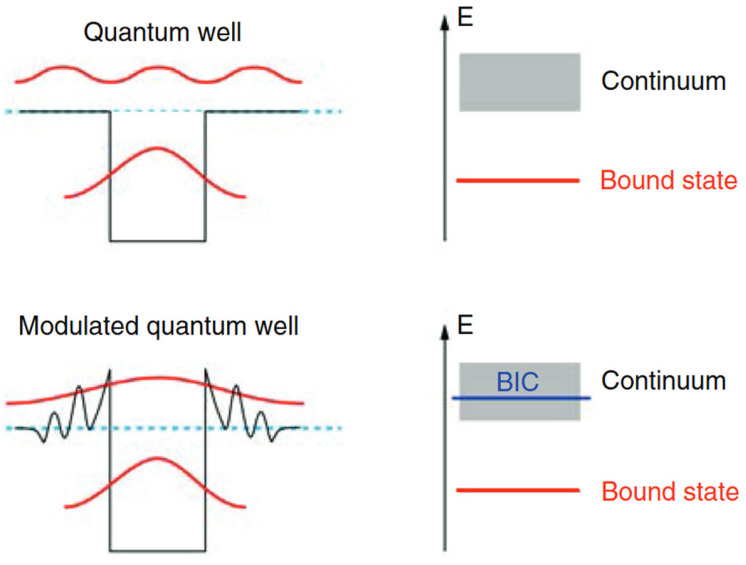
Schematic illustration of the origin of BICs in quantum mechanical systems [4].

In the fields of quantum optics and quantum information science, the traditional understanding holds that within a closed system, light cannot enter the radiation channel and can only exist stably in a bound state; however, if it is in an open system, it will couple with the external continuum, preventing it from maintaining the bound state. Instead, it can only exist in a resonant mode, resulting in energy leakage and radiation to infinity [1]. However, with the development of the investigations, the unique phenomenon of bound states in the continuum (BIC) has been found and verified. In 1929, John von Neumann and Eugene Wigner, based on the Schrödinger equation in the field of quantum mechanics, proposed this concept, which became an unconventional phenomenon derived from quantum mechanics [2]. They pointed out that although BIC is in the continuous domain, it has the ability to avoid interacting with the surrounding continuous spectrum, thereby achieving zero energy radiation locally in the continuous spectrum of radiation waves and becoming a perfect bound state [3]. The energy spectrum of a common quantum well includes oscillating non-local states in the continuum and discrete bound states within the potential well. However, in the three-dimensional quantum potential well artificially designed by John von Neumann and Eugene Wigner, it was discovered that by modulating the potential well, it is possible to achieve perfect localization around the potential well in the continuous domain, as shown in Figure 1. 

As the research expands, BIC has been widely studied in various fields of wave physics, including electromagnetic waves [5], aero-acoustic waves [6], water waves [7], and elastic waves in solids [8]. Due to the highly limited optical properties, BIC shows many unique properties when the optical field is confined to a tiny spatial scale, including enhancing the light–matter interaction, effectively suppressing the radiation loss, and theoretically having a vanishing resonance linewidth and an infinite quality factor (*Q*-factor) [4,9]. However, it is constrained by practical conditions, such as structural defects, material absorption, and size limitations [10,11]. The linewidth of ideal BIC tends to be infinitesimal, which makes it difficult for experimental instruments to directly detect [12]. To solve this problem, external disturbances are usually introduced into the design [13,14]; the structural symmetry is broken by adjusting the geometric shape and structural parameters (translation, rotation, and size change) of the BIC structure or changing the incident angle [15], and the ideal BIC is converted into a measurable quasi-BIC (qBIC) [11]. It exhibits an asymmetric, extremely sharp, and narrow Fano resonance peak in the spectrum. The formation mechanism of qBIC is that the continuous domain and discrete state are coupled to achieve interference cancellation and convert the non-radiative bound mode into a leaky mode [16]. On the basis of ensuring the detectability, the *Q*-factor of qBIC can be flexibly tuned by changing the breaking parameters to achieve high *Q*-factor performance. Because qBIC has the advantages of a high *Q*-factor and adjustable *Q*-factor, it shows great potential in many applications, including nonlinear optics [17,18], optical switches [19], low-threshold lasers [20,21], ultra-sensitive sensors [22,23], narrowband filters [24,25], etc.

For high-performance refractive index sensors, a high *Q*-factor is very important. It can detect the small frequency shift caused by the small size of the analyte or weak environmental changes [10]. Recently, metal–dielectric hybrid nanostructure sensors based on qBIC have attracted extensive attention in the field of metasurfaces. Compared with traditional plasmonic sensors, these sensors exhibit higher sensitivity and a stronger field enhancement effect at the interface, thanks to the inclusion of plasmonic components. This overcomes the absorption loss problem existing in plasmonic sensors [26]. At the same time, due to their advantages of a high refractive index sensitivity of metals and high *Q*-factor of dielectrics [12,27]. The metal–dielectric hybrid nanostructures effectively reduced the loss, and they have a high figure of merit (*FOM*). Specifically, the reduction in loss includes two parts: one is the radiation loss of the resonant cavity, and the other is the material loss of the resonant cavity. When the loss decreases, the *Q*-factor increases and the linewidth narrows, which not only improves the performance of the metasurface but also enhances the efficiency of the electromagnetic response. However, it is limited by the complexity of the process, stability, and detection range.

Among the many metasurfaces, the tetramer structure shows unique advantages, which can effectively enhance the light–matter interaction, resulting in higher surface sensitivity and polarization insensitivity. In addition, compared with other structures, this structure has a larger size wavelength ratio, which makes it more advantageous in the actual preparation process [11]. In terms of shape selection, a cylindrical shape is a better low-cost sensing platform than other shapes and is applicable to high-precision nanofabrication techniques [22]. The hemispherical design at the top can further increase the contact area between light and matter, thereby enhancing the light absorption. Si_3_N_4_ is a high refractive index dielectric material, which usually has high transparency, good light transmittance, and excellent thermal stability in the visible to near-infrared bands. It can maintain the performance well under high-temperature conditions and has low scattering loss, which is helpful to improve the sensitivity and signal-to-noise ratio of optical sensors and significantly enhance the ability of the metasurface to confine light [28]. Gold has good chemical stability and biocompatibility. It can support the formation of the surface plasmon (SP) effect. Through the SP effect, ultra-high sensitivity detection can be achieved [29].

This paper proposes a metal–dielectric yurt tetramer metasurface based on Fano resonance in the visible to near-infrared bands. By using the finite-difference time-domain (FDTD) method and multipole decomposition in the Cartesian coordinate system, the physical mechanism of resonance modes was investigated, and the effects of size parameters, period, incident light, and refractive index of the surrounding medium on the refractive index sensitivity, *FOM*, and *Q*-factor were quantitatively analyzed.

## 2. Models and Methods

In this paper, the refractive index sensing properties of the metal–dielectric yurt tetramer metasurface are simulated by using the FDTD method. The reflection spectrum, refractive index sensitivity, *FOM*, and *Q*-factor of the metasurface are calculated.

### 2.1. Model and Modeling

**Figure 2:**
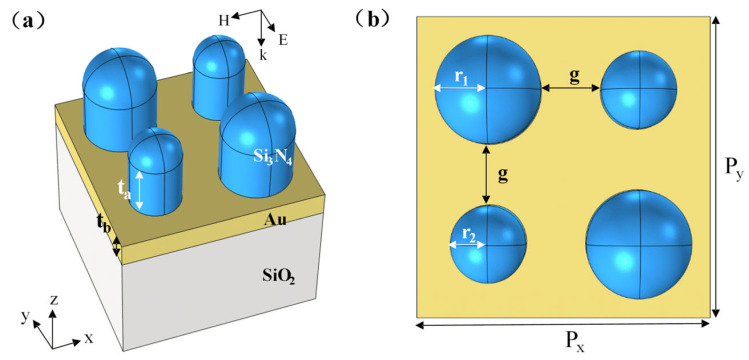
Unit structure of the metal–dielectric yurt tetramer metasurface. (**a**) Three-dimensional schematic diagram; (**b**) *xoy* plane schematic diagram.

**Figure 3:**
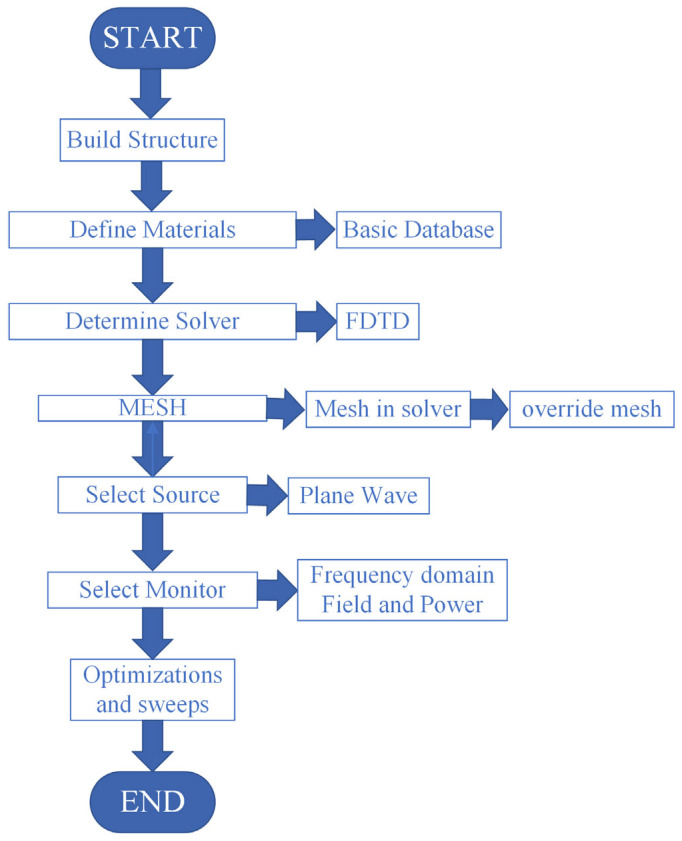
The operation procedure of the FDTD software.

Figure 2 shows the unit structure model of the metal–dielectric yurt tetramer metasurface proposed in this paper. The proposed structure is built upon a semi-infinite thickness SiO_2_ substrate, a gold (Au) layer deposited on it, and a tetramer composed of a Si_3_N_4_ cylinder and a Si_3_N_4_ hemisphere. The initial parameters are set as follows: The top Si_3_N_4_ hemisphere radius *r*_1_ is 140 nm, *r*_2_ is 100 nm, the Si_3_N_4_ cylinder height *t*_a_ is 225 nm, and the Au layer thickness *t*_b_ is 100 nm. The period of the unit structure in the *x* and *y* directions is 840 nm, the spacing between adjacent unit *g* is 160 nm, and the two Si_3_N_4_ composite structures on each diagonal are the same size. Nanomaterials of noble metals (Au, Ag, Cu), due to surface plasmon resonance (SPR) in the visible light band, are widely used in plasmon sensors. Their performance depends on the radiation and absorption efficiency of plasmons [30]. When simulating using software such as Ansys Lumerical FDTD 2023 R2.1, due to the neglect of quantum corrections, there are coupling effects between plasmons at the nanoscale and semiconductor band electrons, as well as significant enhancement of plasmon damping, which can lead to low efficiency of the simulated plasmon-induced effect [31]. But when light interacts with metal nanoparticles whose size is greater than the average free path of free electrons, the dielectric function will not be affected by the size of the nanoparticles. So, we did not take into account the quantum effects involved, based on the above considerations. The refractive indices of the SiO_2_ substrate and Si_3_N_4_ are from the refractive index database [32]; the refractive index of Au was fitted from the discrete data in Palik’s *Handbook of Optical Constants of Solids* into continuous values suitable for simulation; and the refractive index of the surrounding environment was set to 1.333. In order to investigate the reflection spectrum properties and resonance physical mechanism of the metasurface, all simulations are based on the Ansys Lumerical FDTD 2023 R2.1. In the simulation process, periodic boundary conditions (PBC) are applied to the metasurface in the *x* and *y* directions, used to simulate the solution spreading to infinity, and the perfectly matched layer (PML) is used in the *z* direction to ensure that all plane waves incident from any angle onto the absorption layer area through the FDTD free space are completely absorbed without any reflection. The grid spacing in *x*, *y*, and *z* directions is uniformly set at 10 nm, and only TE polarization with an electric field parallel to the *y* direction is considered. When the incident electromagnetic wave is perpendicularly incident along the negative *z*-axis in the *xoz* plane, the structure will show specific optical response properties. The operation procedure is shown in Figure 3. First, build the model and set the required location and materials. Then, set the simulation area and boundary conditions. Next, set the perform meshing. After that, set up the light source. Finally, after checking the material fitting and memory, you can click the run.

### 2.2. Surface Plasmon Resonance Theory

When light interacts with the electrons on the metal surface, elementary excitations are produced, which are called SP. It can produce two types of resonance phenomena; one is called SPR, and the other is called localized surface plasmon resonance (LSPR).

SPR refers to the phenomenon where when electrons interact with the surface of a metal nanostructure, the free electrons within the metal nanostructure are simultaneously excited by an external field, thereby giving rise to a series of entirely new optical properties. The surface plasmons excited at the interface of the metal medium at this moment are called surface plasmon polaritons (SPPs) [33,34]. LSPR refers to the resonance phenomenon that occurs when the vibration of free electrons in a metal nanostructure is restricted by the size of the structure. The surface plasmons excited at the interface of the metal medium at this time are called localized surface plasmons (LSPs). The research on plasma resonance centered around the collective oscillation of electrons on the metal surface is specifically dedicated to addressing how to effectively confine the electromagnetic field within a certain wavelength range and even further compress it to a sub-wavelength scale below the wavelength.

Under the SPP mode, the electromagnetic field energy is highly concentrated near the interface between the metal and the dielectric, reaching its maximum value at the interface. Due to the metal’s loss, the energy is dissipated, causing the electromagnetic field energy to be closer to the dielectric side and exhibiting exponential decay in the direction perpendicular to the interface.

By solving the Maxwell equations under approximate boundary conditions, the dispersion relation of SPP can be obtained:

(1) $$k_{spp}=k_{0}\sqrt{\frac{\varepsilon_{m}\varepsilon_{d}}{\varepsilon_{m}+\varepsilon_{d}}},$$

where *k_spp_* represents the wave vector of SPP, and *k*_0_ represents the wave vector of the incident light wave. *ε_m_* and *ε_d_* represent the dielectric constants of the metallic material and the dielectric material, respectively.

In terms of material selection, the wavelength and propagation distance of SPP are two important characteristic parameters. The wavelength of SPP *λ_spp_* can be calculated from the real part of its wave vector, and the propagation distance of SPP *δ_spp_* mainly depends on the imaginary part of its wave vector:

(2) $$\lambda_{spp}=\frac{2\pi }{k_{spp}^{\prime }}=\sqrt{\frac{\varepsilon_{d}+\varepsilon_{m}^{\prime }}{\varepsilon_{d}\varepsilon_{m}^{\prime }}},$$

(3) $$\delta_{spp}=\frac{1}{2k_{spp}^{\prime \prime }}=\lambda_{0}\frac{\varepsilon_{m}^{\prime 2}}{2\pi \varepsilon_{m}^{\prime \prime }}\sqrt{\frac{\varepsilon_{d}+\varepsilon_{m}^{\prime }}{\varepsilon_{d}\varepsilon_{m}}},$$

We require a longer transmission distance and higher far-field transmission efficiency. Therefore, we need low-loss metal materials with large negative real parts and small imaginary parts of the metal dielectric constant, and we also need to make the dielectric constants of the media on both sides of the metal film as close as possible. These will lead to better physical phenomena.

### 2.3. The Key Indicators of Refractive Index Sensing

When evaluating the performance of a refractive index sensor, refractive index sensitivity, *Q*-factor, and *FOM* are commonly used as key indicators. Among them, the bulk refractive index sensitivity (*S_bulk_*) is one of the widely used sensing indicators, which is used to measure the ability of the structure to sense the changes of refractive index of the environment in the whole region. It is defined as the change of resonance wavelength caused by the change of refractive index unit (RIU):

(4) $$S_{bulk}=\frac{\Delta \lambda_{res}}{\Delta n},$$

where Δ*n* represents the change in environmental refractive index, and Δ*λ_res_* represents the change in resonance wavelength with Δ*n*.

The *Q*-factor is an important parameter that describes the loss of resonance systems. It is commonly used to measure the stability of sensor performance. A high *Q*-factor indicates that the system has lower energy loss and can respond more sensitively to changes in the detection environment. The definition of *Q*-factor is as follows:

(5) $$Q=\frac{\lambda_{res}}{\mathrm{FWHM}},$$

where *λ_res_* represents the wavelength corresponding to the resonance position, and full width at half maximum (FWHM) represents the spectral linewidth at half the height of the resonance peak.

In addition, a higher *FOM* can achieve a high signal-to-noise ratio for resonance wavelength shift measurement, thereby enabling high-performance refractive index sensors that can be used to evaluate the comprehensive resolution capability of optical sensors. *FOM* includes *S_bulk_* and *Q*-factor, which can be defined as the ratio of *S_bulk_* to FWHM,

(6) $$FOM=\frac{S_{bulk}}{\mathrm{FWHM}}.$$

## 3. Results and Discussion

The refractive index sensing properties of metasurfaces are easily affected by factors such as size, period, light source, and surrounding environment. To deeply investigate the influence of these factors on the refractive index sensing properties, this work takes the metal–dielectric yurt tetramer metasurface as the research object, analyzes the generation mechanism of the resonance modes, and gives the significant correlation between the period, size (height and radius) of the Si_3_N_4_ cylinders, surrounding environment, incident light, asymmetry of the Si_3_N_4_ cylinder, and the refractive index sensing properties. Except for the analysis of the influence of environmental refractive index on refractive index sensing, the refractive index of the surrounding environment in all other analyses is 1.333.

### 3.1. Resonance Modes Analysis of Metal–Dielectric Yurt Tetramer Metasurface

**Figure 4:**
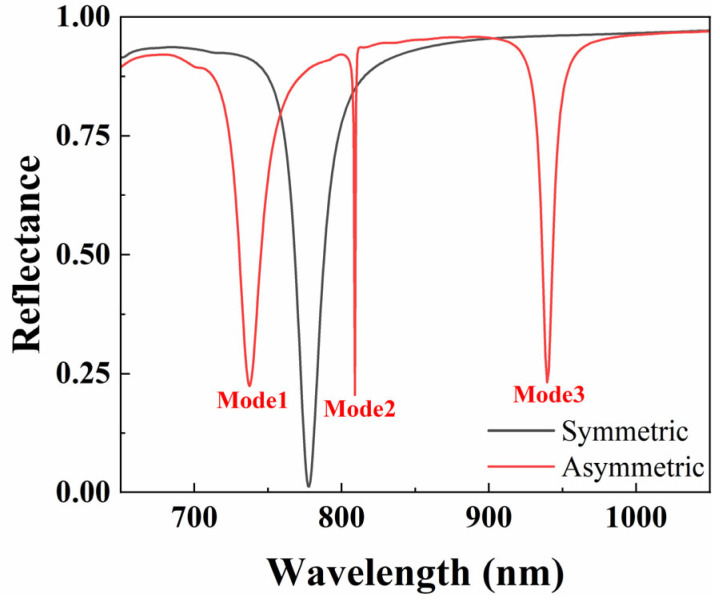
Comparison of reflectance spectra of symmetric and asymmetric structures (for a symmetric structure, *r*_1_ = *r*_2_ = 140 nm, *P* = 840 nm, *t*_a_ = 225 nm, and *t*_b_ = 100 nm).

The reflection spectra of symmetric and asymmetric structures are shown in Figure 4. The results show that the asymmetric structure excited three distinct resonance peaks in the reflection spectrum, corresponding to three different resonance modes. The appearance of these resonance peaks can be attributed to the qBIC modes introduced by changing the size of the diagonal yurt to break the in-plane inversion symmetry of the unit cell. In the symmetrical structure, some resonance modes become perfect BICs due to symmetry protection, and these modes theoretically do not radiate energy into free space, so there will be no obvious resonance peaks appearing in the reflection spectrum. However, through symmetry breaking (such as introducing structural size perturbations), these symmetric protected BIC modes are transformed into qBIC modes. Although the qBIC modes still have a high *Q*-factor, they allow limited energy to leak into free space, resulting in the formation of distinct resonance peaks in the reflected spectrum. The emergence of Mode 2 and Mode 3 is precisely the result of this symmetry-breaking effect [10].

**Figure 5:**
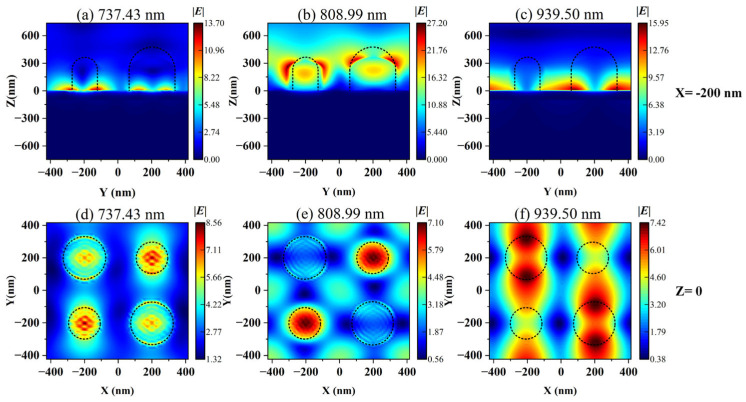
The distribution of simulated electric field amplitude |***E***|. (**a**) The electric field distribution of Mode 1 in the *yoz* plane; (**b**) The electric field distribution of Mode 2 in the *yoz* plane; (**c**) The electric field distribution of Mode 3 in the *yoz* plane; (**d**) The electric field distribution of Mode 1 in the *xoy* plane; (**e**) The electric field distribution of Mode 2 in the *xoy* plane; (**f**) The electric field distribution of Mode 3 in the *xoy* plane.

**Figure 6:**
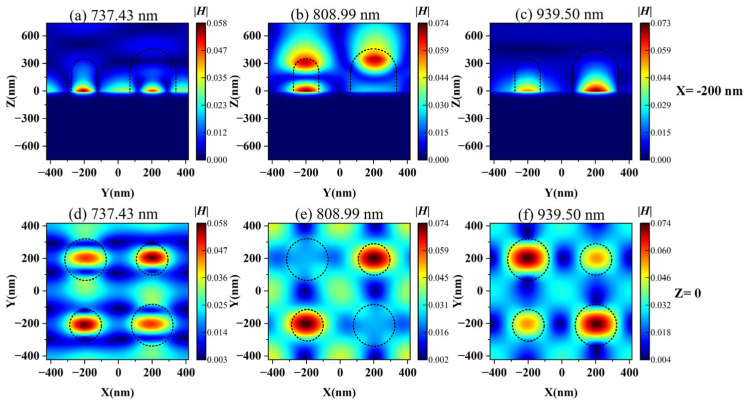
The distribution of simulated magnetic field amplitude |***H***|. (**a**) The magnetic field distribution of Mode 1 in the *yoz* plane; (**b**) The magnetic field distribution of Mode 2 in the *yoz* plane; (**c**) The magnetic field distribution of Mode 3 in the *yoz* plane; (**d**) The magnetic field distribution of Mode 1 in the *xoy* plane; (**e**) The magnetic field distribution of Mode 2 in the *xoy* plane; (**f**) The magnetic field distribution of Mode 3 in the *xoy* plane.

We further calculated the distribution of electric field (Figure 5) and magnetic field (Figure 6) corresponding to different resonance wavelengths for three modes under asymmetric structures. It can be seen from Figure 5a,d, as well as Figure 6a,d, that Mode 1 is mainly dominated by the SPR effect, and its electromagnetic field is significantly enhanced near the interface between the metal and the dielectric, exhibiting a highly localized effect. Mode 2 and Mode 3 correspond to dielectric-dominated qBIC (d-qBIC) and metal-dominated qBIC (m-qBIC), respectively.

In Mode 2 (see Figure 5b,e and Figure 6b,e), the electromagnetic field exhibits a uniform and widespread distribution inside and at the edges of the medium. In Mode 3 (see Figure 5c,f and Figure 6c,f), the electromagnetic field is significantly enhanced near the interface between the metal and the dielectric and partially extends to the dielectric layer. The common feature of these modes is the ability to confine most of the electric and magnetic fields within low-loss metasurfaces, thereby achieving significant localized field enhancement effects. This indicates that this structure can effectively enhance the interaction between matter and incident waves, improve sensing performance, and have excellent bulk sensing capabilities.

**Figure 7:**
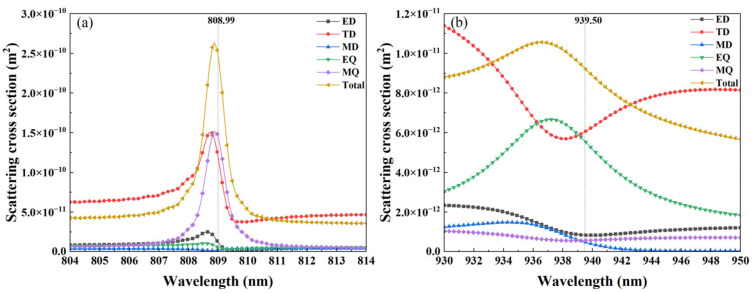
Multipole decomposition in the Cartesian coordinate system. (**a**) Multipole decomposition at the resonance wavelength of Mode 2; (**b**) Multipole decomposition at the resonance wavelength of Mode 3.

To deeply explore the causes of the generation of these two Fano resonance modes, we used multipole decomposition in Cartesian coordinates to calculate the contributions of electric dipole (ED), magnetic dipole (MD), toroidal dipole (TD), electric quadrupole (EQ), and magnetic quadrupole (MQ) to the resonance of Modes 2 and 3. The decomposition calculation formulas of the multipole scattering power are listed in Table 1. In the table, $\vec{J}=-i\omega \varepsilon_{0}(\varepsilon_{r}-1)\vec{E}$ in the Cartesian coordinate system $\vec{J}$ represents the polarization current at the internal point *r* = (*x*, *y*, *z*), *ω* is angular frequency, *ε*_0_ is the permittivity of vacuum, *ε_r_* is the relative permittivity of the material, and $\vec{E}$ is the E-field in the scatterer. Subscripts *α*, *β*, and *γ* represent the Cartesian axes *x*, *y*, or *z*. At *λ* = 808.99 nm (Mode 2), as shown in Figure 7a, the scattering intensity of the MQ moment exceeds that of other multipoles, indicating that Mode 2 is mainly contributed by MQ within a wide wavelength range, followed by TD, and other multipolar components are significantly suppressed. At *λ* = 939.50 nm (Mode 3), Figure 7b shows that the scattering intensity of the TD moment dominates, indicating that Mode 3 is mainly excited by the TD qBIC resonance with a high *Q*-factor, while the EQ moment plays a secondary role, and the remaining multipolar components are also strongly suppressed.

### 3.2. Effect of Period on Refractive Index Sensing

**Figure 8:**
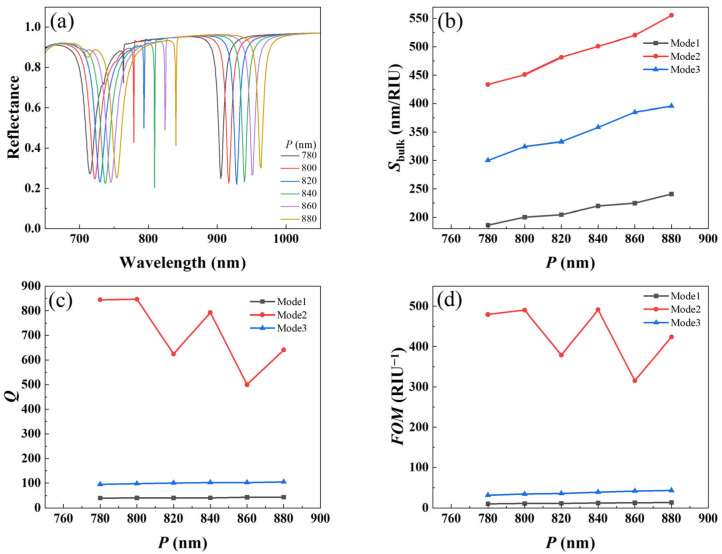
(**a**) Reflection spectra of metal–dielectric yurt tetramer metasurface; (**b**) *S_bulk_*; (**c**) *Q*-factor; and (**d**) *FOM* as a function of *P* (where *r*_1_ = 140 nm, *r*_2_ = 100 nm, *t*_a_ = 225 nm, and *t*_b_ = 100 nm).

In order to quantitatively investigate the effect of the period of structure on the refractive index sensing, we calculated the reflection spectra of metal–dielectric yurt tetramer metasurfaces with periods of 780 nm, 800 nm, 820 nm, 840 nm, 860 nm, and 880 nm, respectively, as shown in Figure 8a. It can be seen that with the period increases, the distance between the unit structures widens, which alters the light scattering and interference of the structure, thereby causing the resonance wavelength to shift towards a longer wavelength. In summary, the resonance wavelength of the metal–dielectric yurt tetramer metasurface gradually red shifts with increasing period. Figure 8b–d show that in Mode 2, *S_bulk_*, *Q*-factor, and *FOM* are relatively large, and *S_bulk_* gradually increases with the increase of period *P*. Based on the trend of *Q*-factor and *FOM* variation with *P*, this paper chooses *P* at 840 nm as the compromise solution. When *P* is 840 nm, Mode 2 (resonance wavelength of 808.99 nm) has a narrow FWHM, indicating that a higher *Q*-factor is obtained here. The *Q* value calculated by Equation (2) is 793.13. According to the formula *R*′ = [(*R*_peak_ − *R*_antipeak_)/(*R*_peak_ + *R*_antipeak_)] × 100% (where *R*′ is the modulation depth, *R*_peak_ is the reflectivity at the Fano resonance peak, and *R*_antipeak_ is the reflectivity at the Fano resonance valley), the modulation depth of the resonance peak is calculated to be about 64%. This modulation depth makes high-*Q* resonance easier to observe and detect in practical applications and has a high resonance signal-to-noise ratio during actual detection.

### 3.3. Effect of Size on Refractive Index Sensing

#### 3.3.1. Effect of Radius of Large Cylinder

**Figure 9:**
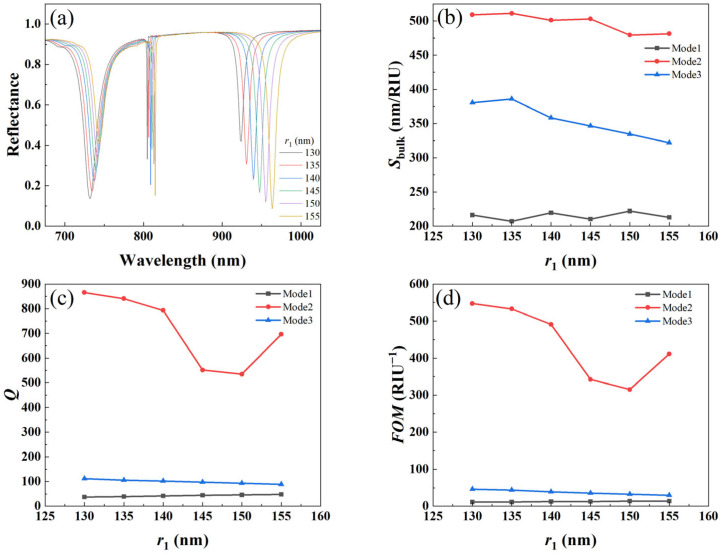
(**a**) Reflectance spectrum of the metal–dielectric yurt tetramer metasurface; (**b**) *S_bulk_*; (**c**) *Q*-factor; and (**d**) *FOM* vary with *r*_1_ (where *P* = 840 nm, *r*_2_ = 100 nm, *t*_a_ = 225 nm, and *t*_b_ = 100 nm).

In order to quantitatively investigate the effect of different radii of the large cylinder *r*_1_ on the refractive index sensing, we calculated the reflection spectra of the metal–dielectric yurt tetramer metasurface with *r*_1_ values of 130 nm, 135 nm, 140 nm, 145 nm, 150 nm, and 155 nm, respectively, as shown in Figure 9a. It can be seen that as the radius *r*_1_ increases, the resonance wavelength of the metal–dielectric yurt tetramer metasurface gradually undergoes a red shift, which is attributed to the increase in the proportion of the internal field distribution within the metasurface. And as *r*_1_ increases, the modulation depth of Mode 3 gradually increases, while the modulation depth of Mode 1 gradually decreases. This is because as *r*_1_ increases, the scattering process of light in the structure is enhanced, resulting in a longer propagation path of light and causing the resonance wavelength to shift towards longer wavelengths. Figure 9b–d show the variation trends of *S_bulk_*, *Q*-factor, and *FOM* size with parameter *r*_1_ in three modes. It can be seen that in Mode 2, the values of *S_bulk_*, *Q*-factor, and *FOM* are relatively large. Among them, the *Q*-factor and *FOM* of Mode 2 decrease first and then increase. Based on the modulation depth of Mode 2 and three sensing indicators, *r*_1_ was ultimately selected as 140 nm.

#### 3.3.2. Effect of Radius of Small Cylinder

**Figure 10:**
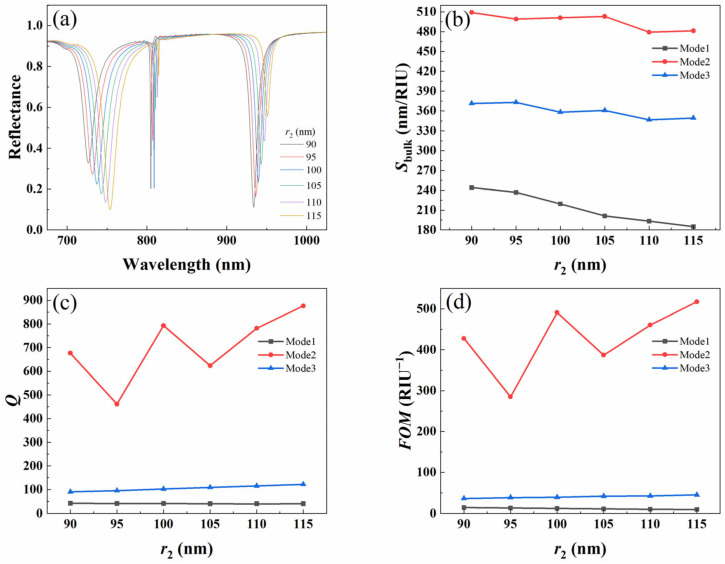
(**a**) Reflectance spectrum of the metal–dielectric yurt tetramer metasurface; (**b**) *S_bulk_*; (**c**) *Q*-factor; and (**d**) *FOM* as a function of *r*_2_ (where *P* = 840 nm, *r*_1_ = 140 nm, *t*_a_ = 225 nm, and *t*_b_ = 100 nm).

In order to quantitatively analyze the effect of different small cylinder radius *r*_2_ on the refractive index sensing, we calculated the reflection spectra of metal–dielectric yurt tetramer metasurface with *r*_2_ values of 90 nm, 95 nm, 100 nm, 105 nm, 110 nm, and 115 nm, respectively. The results are shown in Figure 10a. As the radius *r*_2_ increases, the resonance wavelength shows a red shift trend, and the modulation depth of Mode 1 gradually increases, while the modulation depth of Mode 3 gradually decreases. Further analysis shows that with the change of *r*_2_, the *S_bulk_*, *Q*-factor, and *FOM* under the three modes exhibit different trends of change, as shown in Figure 10b–d. Especially in Mode 2, the values of *S_bulk_*, *Q*-factor, and *FOM* are relatively high. Based on the modulation depth of Mode 2 and three sensing indicators, *r*_2_ was ultimately selected as 100 nm.

#### 3.3.3. Effect of Cylinder Height

**Figure 11:**
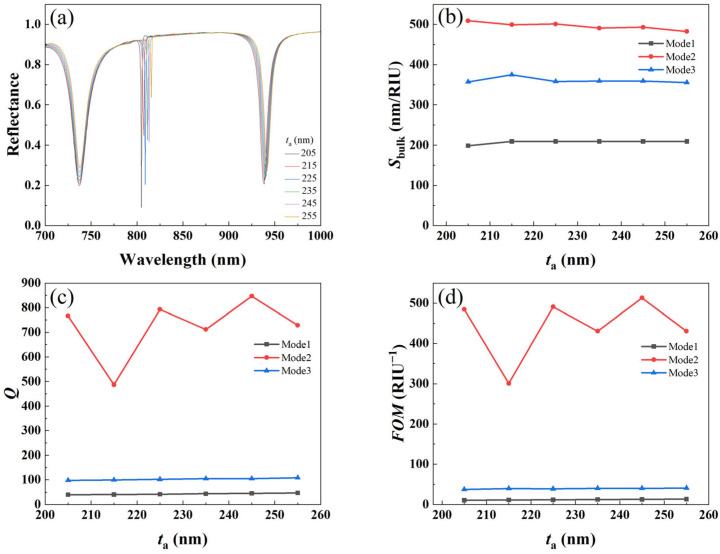
(**a**) Reflectance spectra; (**b**) *S_bulk_*; (**c**) *Q*-factor; and (**d**) *FOM* of the metal–dielectric yurt tetramer metasurface as a function of *t*_a_ (where *P* = 840 nm, *r*_1_ = 140 nm, *r*_2_ = 100 nm, and *t*_b_ = 100 nm).

In order to quantitatively analyze the effect of different cylinder heights *t*_a_ on the refractive index sensing, we calculated the reflection spectra of metal–dielectric yurt tetramer metasurfaces with *t*_a_ values of 205 nm, 215 nm, 225 nm, 235 nm, 245 nm, and 255 nm, respectively. The results are shown in Figure 11a. From Figure 11a, it can be seen that as the height increases, the resonance wavelength of Mode 1 remains unchanged. This is because Mode 1 is caused by the SPR mode, and it is independent of changes in the height of the medium. However, the resonance wavelengths of Modes 2 and 3 red shift due to the close similarity between qBIC modes and waveguide mode in the optical field. As the height increases, the relative effective volume occupied by the waveguide mode will correspondingly increase, leading to an increase in the effective refractive index of the waveguide mode. According to the effective refractive index formula *n*_eff_ = *β*/*k*_0_ (among them, *β* is the propagation constant and *k*_0_ = 2π/*λ* is the wavenumber in vacuum), the resonance position will red shift. Figure 11b–d show the variation of *S_bulk_*, *Q*-factor, and *FOM* with size parameter *t*_a_ in three modes. It can be seen that *S_bulk_*, *Q*-factor, and *FOM* of Mode 2 have significantly larger values. Based on the modulation depth required for Mode 2 and the advantages of three sensing indicators, we selected *t*_a_ as 225 nm. Taking into account the above variation patterns, the structure designed in this work will focus on Mode 2, as it exhibits better refractive index sensing properties.

### 3.4. Effect of Refractive Index of Environment on Refractive Index Sensing

**Figure 12:**
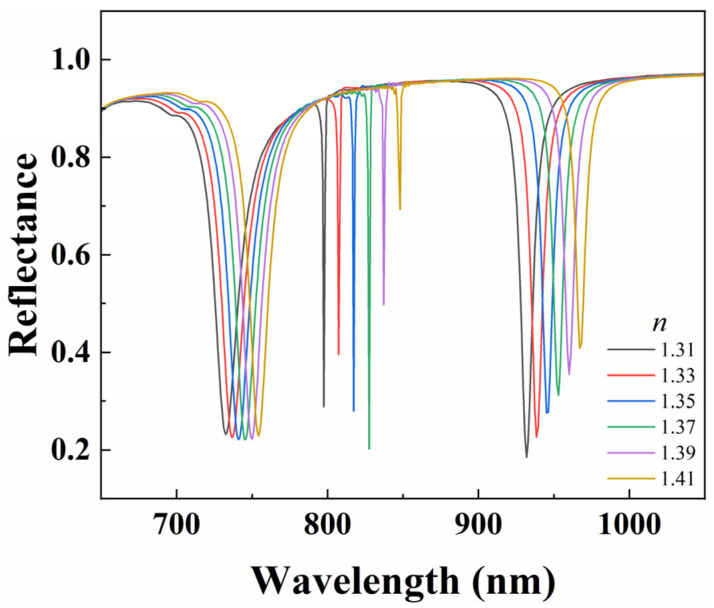
Reflectance spectra of metal–dielectric yurt tetramer metasurface as a function of *n* (where *P* = 840 nm, *r*_1_ = 140 nm, *r*_2_ = 100 nm, *t*_a_ = 225 nm, and *t*_b_ = 100 nm).

The resonance wavelength of the sensor is closely related to the refractive index of the surrounding environment. When the analyte adheres to the structure, the refractive index of the environment changes, thereby causing a shift in the resonance wavelength. Therefore, biosensors can detect the presence and quantity of analytes by monitoring spectral shifts caused by subtle changes in environmental refractive index [36,37,38]. The design of sensors for biosensing can be realized by using this property. To evaluate the performance of the metasurface sensor in measuring environmental refractive index changes, we investigate the effect of surrounding media with different refractive indices *n* (1.31, 1.33, 1.35, 1.37, 1.39, and 1.41) on Fano resonance. As shown in Figure 12, the resonance wavelength gradually red shifts with the increase of environmental refractive index, but the overall shape of Fano resonance is not affected, except that the modulation depth of the Fano peak suddenly decreases in a particular case. This indicates that the change in environmental refractive index has no significant effect on the linearity and *FOM* of Fano resonance [39]. In addition, the modulation depth of Mode 1 increases first and then decreases, while the modulation depth of Mode 3 gradually decreases. This indicates that this metasurface has achieved effective sensing performance. Therefore, changes in the surrounding environment can be quantitatively measured by detecting changes in resonance wavelength. By observing the shift of resonance wavelength under vertically incident electromagnetic wave irradiation and further analyzing the variation of spectral shift with refractive index, the sensitivity of the metasurface sensor can be determined.

### 3.5. Effect of Incident Light on Refractive Index Sensing

**Figure 13:**
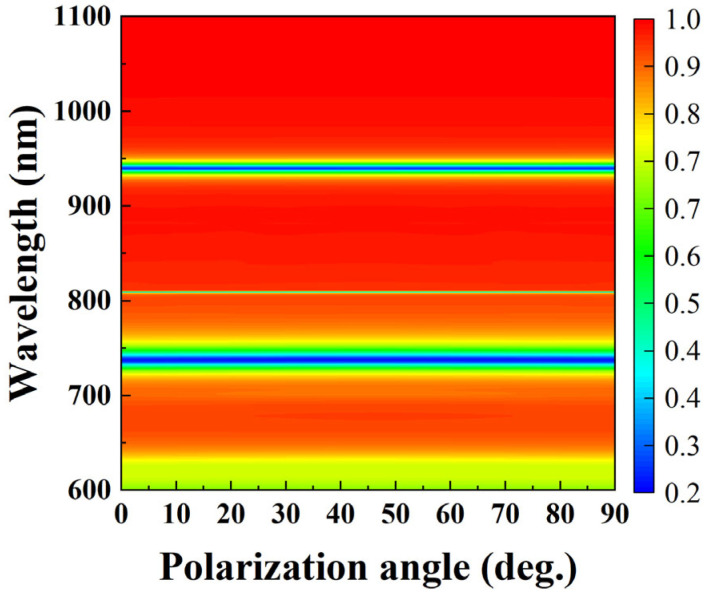
Reflectance spectra of metal–dielectric yurt tetramer metasurface as a function of polarization angle (where *P* = 840 nm, *r*_1_ = 140 nm, *r*_2_ = 100 nm, *t*_a_ = 225 nm, and *t*_b_ = 100 nm).

Biosensors with polarization dependence are affected by biomolecules and sensitivity to light source conditions, which limits their practical applications [40]. In addition, such sensors are sensitive to changes in the external environment, which may affect the accuracy and stability of the sensor [41]. Meanwhile, polarization dependence may increase costs and size, which is unfavorable for the development of portable instruments. Figure 13 shows the reflection spectra of the metal–dielectric yurt tetramer metasurface as a function of polarization angle. From Figure 13, it can be seen that when the polarization angle of the vertically incident electromagnetic wave is changed, the position and spectral width of the reflection resonance valley remain almost unchanged, indicating the polarization insensitivity of the structure. Therefore, the polarization of the metasurface is independent, ensuring that they can effectively respond to incident electromagnetic waves regardless of the polarization state.

**Figure 14:**
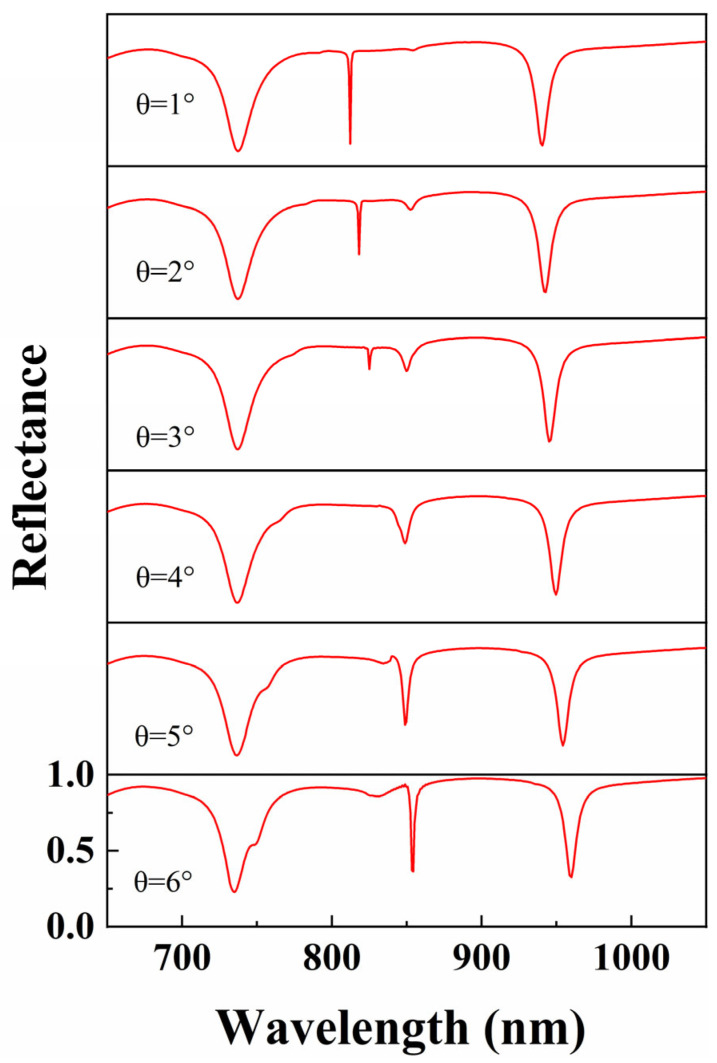
Reflectance spectra of metal–dielectric yurt tetramer metasurface as a function of incident angle (where *P* = 840 nm, *r*_1_ = 140 nm, *r*_2_ = 100 nm, *t*_a_ = 225 nm, and *t*_b_ = 100 nm).

Next, we simulated the reflection spectra of the metal–dielectric yurt tetramer metasurface as the incident angle of light varied from 1° to 6°, with a step size of 1°. The simulation results are shown in Figure 14. It was found that the peak of Fano resonance shifted with the change of incident angle, and the modulation depth varied. Specifically, as the incident angle increases, the resonance wavelength of Mode 1 blue shifts, while the resonance wavelength of Mode 3 red shifts. The resonance valley of Mode 2 splits first and then couples. The ideal BIC will be excited at a specific incident angle and collapse into qBIC as the incident angle increases or decreases.

### 3.6. Effect of Asymmetry on Refractive Index Sensing

**Figure 15:**
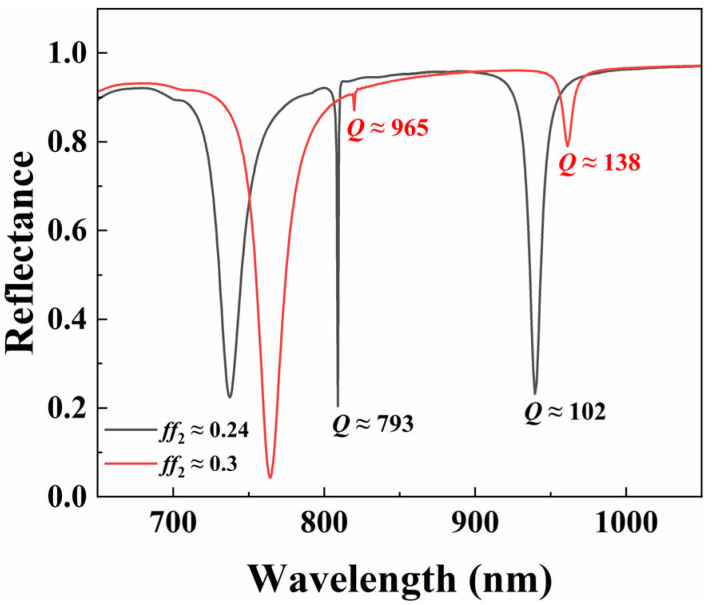
Reflectance spectra at *ff*_2_ ≈ 0.24 (black solid line) and *ff*_2_ ≈ 0.3 (red solid line).

In order to quantify the degree of structural asymmetry, asymmetric parameters *ff*_1_ and *ff*_2_ were introduced (*ff*_1_ = 2*r*_1_/*P* and *ff*_2_ = 2*r*_2_/*P*), with a range of 0–1. Figure 15 shows the reflection spectra of a nearly symmetric structure (asymmetric parameter *ff*_2_ ≈ 0.3) and an asymmetric structure (asymmetric parameter *ff*_2_ ≈ 0.24). Among them, due to the dominant dielectric properties, the d-qBIC mode exhibits a higher *Q*-factor; due to the dominant plasmonic properties, m-qBIC exhibits a lower *Q*-factor. From Figure 15, it can be seen that the symmetrical protection BIC corresponding to the nearly symmetrical structure with ultra-narrow linewidth has a high *Q*-factor. However, due to the overly ideal structure and insufficient radiation leakage, the modulation depth is too small, resulting in a poor signal-to-noise ratio of the resonance during actual detection, which is difficult to detect in practical applications. In view of this, we used the asymmetric parameter *ff*_2_ ≈ 0.24, thereby obtaining a larger *Q*-factor of approximately 793.

This work conducted structural optimization based on the metasurface proposed in reference [10]. By comparing the refractive index sensing performance parameters of various metasurfaces in the visible to near-infrared spectral range (see Table 2), it was found that the structure designed in this paper performs excellently in terms of *FOM* and *Q*-factor. Although the sensitivity is slightly lower than that reported in reference [10], its overall performance is still better than other metasurfaces in the literature. Therefore, this structure shows great potential applications in the field of optical sensing. It can achieve more accurate refractive index detection with high sensitivity, high resolution, and low energy loss. Thus, this structure will play an important role and have value in the fields of biomolecule detection and environmental monitoring.

## 4. Conclusions

This paper designs a metal–dielectric yurt tetramer metasurface and investigates the BIC of the metasurface under the TE wave incidence. Using FDTD simulation, it was found that there are three resonance modes in this metasurface, including SPR mode, d-qBIC mode, and m-qBIC mode. Using the multipole decomposition method, the physical mechanisms of the two Fano resonance modes were accurately calculated and revealed. We quantitatively analyzed the effect of different influencing factors on the properties of Fano resonances and carried out detailed numerical calculations on the refractive index sensing properties of metasurface. The results indicated that all three resonance modes exhibit narrow FWHM; the d-qBIC mode has an ultra-narrow FWHM of 1.02 nm. Such a narrow FWHM is beneficial for sensors to detect resonance wavelength shifts caused by small changes in the environment, thereby achieving high-precision detection. In addition, the structure also has a high *Q* value and *FOM*, while exhibiting better wavelength tunability and polarization insensitivity in the visible to near-infrared range. These properties fully demonstrate that metal–dielectric tetramer metasurfaces are an ideal choice for refractive index sensing applications. Specifically, this structure achieved ultra-narrow FWHM through asymmetric modulation and achieved the sensitivity of 500.94 nm/RIU, *FOM* of 491.12 RIU^−1^, and *Q*-factor of 793.13. These excellent performance indicators demonstrate that the structure has important potential applications in the field of optical sensing. It is expected to provide a new sensing scheme with high sensitivity, high resolution, and low loss for practical applications, including biological molecule detection and environmental monitoring.

## Figures and Tables

**Table 1:** The decomposition calculation formulas of the multipole scattering power [35].

Multipoles	Expression	Far Field Scattering Power
Electric dipole (*P*)	$\vec{P}=\frac{1}{i\omega }\int \vec{J}d^{3}\vec{r}$	$I_{p}=\frac{2\omega^{4}}{3c^{3}}{\left|\vec{P}\right|}^{2}$
Magnetic dipole (*M*)	$\vec{M}=\frac{1}{2c}\int (\vec{r}\times \vec{J})d^{3}\vec{r}$	$I_{M}=\frac{2\omega^{4}}{3c^{3}}{\left|\vec{M}\right|}^{2}$
Toroidal dipole (*T*)	$\vec{T}=\frac{1}{10c}\int \left[(\vec{r}\cdot \vec{J})\vec{r}-2r^{2}\vec{J}\right]d^{3}\vec{r}$	$I_{T}=\frac{2\omega^{6}}{3c^{5}}{\left|\vec{T}\right|}^{2}$
Electric quadrupole (*Q_e_*)	$Q_{e}=\frac{1}{i2\omega }\int \left[r_{\alpha }J_{\beta }+r_{\beta }J_{\alpha }-\frac{2}{3}(\vec{r}\cdot \vec{J})\sigma_{\alpha \beta }\right]d^{3}\vec{r}$	$I_{Q}^{e}=\frac{\omega^{6}}{5c^{5}}\Sigma {\left|QE\right|}^{2}$
Magnetic quadrupole (*Q_m_*)	$Q_{m}=\frac{1}{3c}\int \left[{\left(\vec{r}\times \vec{J}\right)}_{\alpha }r_{\beta }+{\left(\vec{r}\times \vec{J}\right)}_{\beta }r_{\alpha }\right]d^{3}\vec{r}$	$I_{Q}^{m}=\frac{\omega^{6}}{20c^{5}}\Sigma {\left|QM\right|}^{2}$

**Table 2:** Comparison of sensing performance parameters of metasurfaces in the visible to near-infrared range.

Source	*FOM* (RIU^−1^)	*Q*-Factor	Sensitivity (nm/RIU)
[42]	445	2000	178
[43]	78	728	161.5
[44]	56.5	133	453.3
[10]	347.4	517.3	510.7
This work	491.12	793.13	500.94

## Data Availability

The original contributions presented in this study are included in the article; further inquiries can be directed to the corresponding authors.
